# Co-Expression Network Analysis and Introgressive Gene Identification for Fiber Length and Strength Reveal Transcriptional Differences in 15 Cotton Chromosome Substitution Segment Lines and Their Upland and Sea Island Parents

**DOI:** 10.3390/plants13162308

**Published:** 2024-08-19

**Authors:** Pengtao Li, Yu Chen, Rui Yang, Zhihao Sun, Qun Ge, Xianghui Xiao, Shuhan Yang, Yanfang Li, Qiankun Liu, Aiming Zhang, Baoguang Xing, Bei Wu, Xue Du, Xiaoyan Liu, Baomeng Tang, Juwu Gong, Quanwei Lu, Yuzhen Shi, Youlu Yuan, Renhai Peng, Haihong Shang

**Affiliations:** 1 School of Biotechnology and Food Engineering, Anyang Institute of Technology, Anyang 455000, China; lipengtao1056@126.com (P.L.); cyu990324@163.com (Y.C.);; 2 National Key Laboratory of Cotton Bio-Breeding and Integrated Utilization, Institute of Cotton Research of Chinese Academy of Agricultural Sciences, Anyang 455000, China; 15388465669@163.com (R.Y.); szhpaxl@163.com (Z.S.); gequn@caas.cn (Q.G.);; 3 National Key Laboratory of Cotton Bio-Breeding and Integrated Utilization, Zhengzhou University, Zhengzhou 450001, China; 4 Xinjiang Production and Construction Corps Seventh Division Agricultural Research Institute, Kuitun 833200, China; 5 Agricultural Technology Popularization Center of Kashgar, Kashgar 844000, China

**Keywords:** cotton chromosome segment substitution lines, fiber quality, weighted gene co-expressed network analyses, functional enrichment analysis, introgressive gene identification

## Abstract

Fiber length (FL) and strength (FS) are the core indicators for evaluating cotton fiber quality. The corresponding stages of fiber elongation and secondary wall thickening are of great significance in determining FL and FS formation, respectively. QTL mapping and high-throughput sequencing technology have been applied to dissect the molecular mechanism of fiber development. In this study, 15 cotton chromosome segment substitution lines (CSSLs) with significant differences in FL and FS, together with their recurrent parental *Gossypium hirsutum* line CCRI45 and donor parent *G. barbadense* line Hai1, were chosen to conduct RNA-seq on developing fiber samples at 10 days post anthesis (DPA) and 20 DPA. Differentially expressed genes (DEGs) were obtained via pairwise comparisons among all 24 samples (each one with three biological repeats). A total of 969 DEGs related to FL-high, 1285 DEGs to FS-high, and 997 DEGs to FQ-high were identified. The functional enrichment analyses of them indicated that the GO terms of cell wall structure and ROS, carbohydrate, and phenylpropanoid metabolism were significantly enriched, while the GO terms of glucose and polysaccharide biosynthesis, and brassinosteroid and glycosylphosphatidylinositol metabolism could make great contributions to FL and FS formation, respectively. Weighted gene co-expressed network analyses (WGCNA) were separately conducted for analyzing FL and FS traits, and their corresponding hub DEGs were screened in significantly correlated expression modules, such as *EXPA8*, *XTH*, and *HMA* in the fiber elongation and WRKY, TDT, and RAC-like 2 during secondary wall thickening. An integrated analysis of these hub DEGs with previous QTL identification results successfully identified a total of 33 candidate introgressive DEGs with non-synonymous mutations between the *Gh* and *Gb* species. A common DEG encoding receptor-like protein kinase 1 was reported to likely participate in fiber secondary cell thickening regulation by brassionsteroid signaling. Such valuable information was conducive to enlightening the developing mechanism of cotton fiber and also provided an abundant gene pool for further molecular breeding.

## 1. Introduction

Cotton is one of the most important commercial crops that is widely cultivated across the world for its textile raw material and edible oil [1]. Cotton fibers are developed from the outer epidermal cells of cotton ovule, and mature seeds developed from ovules accumulate a large amount of oil and protein inside. The contents of oil and protein in mature cotton seeds account for 21% and 23% of the total seed mass, respectively [2]. Cotton fibers play crucial roles in textile industry, of which the production value is directly tied to fiber yield and quality [3]. These facts have led plenty of studies to use multiple methods to investigate the molecular mechanism of fiber development, which is of great theoretical significance and practical value for improving cotton yield and fiber quality.

Cotton fibers are differentiated from single-cell trichomes of ovule epidermises, making them an ideal model for studying cell elongation [4]. Fiber development is an elaborate process which involves multiple regulating pathways and is divided into four different yet overlapping stages, namely the initiation (−3 to 3 days post anthesis, DPA), elongation (0 to 25 DPA), second cell wall biosynthesis (15 to 40 DPA), and maturation (45 to 60 DPA) [5]. Despite all epidermal cells of cotton ovules having the potential to develop into fibers, only 25% to 30% of cells can develop into fibers during the initiation process, which is deemed to be a crucial factor in limiting fiber yield [6]. Therefore, increasing the percentage of ovule epidermal cells differentiating into fibers may be an economically and effective method of increasing fiber yield. Fiber elongation is attributed to the interaction between fiber cell wall relaxation and internal turgor pressure [7], where the latter is mainly from the opening and closing of plasmodesmata and the transport of sucrose or potassium ion [8]. Studies separately using lintless mutants and superior CSSLs to identify the FL-related genes through transcriptome sequencing [9,10,11,12] have shown that multiple transcription factors (TFs), proteins, hormones, fatty acids, lipids, and sugar signaling molecules participate in the regulation of fiber elongation. For example, the bHLH/HLH, MYB, WRKY TFs have been proven to play important roles in fiber development [13,14,15]. Furthermore, the reactive oxygen species (ROS), including H_2_O_2_ and ^−^OH, are found to promote cell expansion via loosening cell walls and regulating cytoskeletons [16]. Certainly, the formation of fiber length could not be accomplished by multiple plant hormones, such as the motivating indole-3-acetic acid (IAA), gibberelline (GA), ethylene (Eth), brassinosteroid (BR), and strigolactone (SL), as well as the inhibiting cytokinin (CK) and abscisic acid (ABA) [17,18,19]. The biosynthesis of secondary cell wall components, including cellulose, hemicellulose (xyglucan and mannose), and lignin, showed a high correlation with its thickness, and these cell wall sediments can improve fiber strength and flexibility [6]. Previous studies have shown that some hormone- and TF-related genes are involved in the development of secondary cell wall thickening, such as *GhGRF4*, *GhERF108*, *GhCes*, *GhARF7*, *GhRAC13* [20,21,22]. After the formation of secondary cell wall, the mineral content of fiber cells continues to decrease, while the cellulose content is maintained at above 90% during the dehydrating stage. However, no fiber maturation-related genes have been reported, and the molecular mechanism of fiber dehydration and maturation still remain indefinable.

Chromosome segment substitution lines (CSSLs) are developed from a single hybridization between the donor and recipient parents, which exhibit significant differences in target traits [23] followed by multiple backcrosses between their filial plants and the recurrent parent, coupled with marker-assisted selection (MAS) [24]. CSSLs can effectively eliminate environmental interference and reduce the influence of the genetic background of a population; therefore, it has been widely utilized for quantitative trait locus (QTL) mapping of cotton fiber yield and quality [25,26]. In this study, developing fibers at 10 DPA and 20 DPA from 15 CSSLs and their parents CCRI45 and Hai1 were separately sampled for transcriptome sequencing, and differentially expressed genes (DEGs) were identified via pairwise comparisons among the 72 samples (including three biological repeats). Gene Ontology (GO) enrichment analysis and Kyoto Encyclopedia of Genes and Genomes (KEGG) annotations of these DEGs were performed to identify their putative biological functions, which were also selected to perform weighted gene co-expressed network analysis (WGCNA) in order to screen the hub DEGs. The introgressive DEGs were predicted by removing the CCRI45-bakground DEGs from the DEG dataset that were specifically expressed between CSSLs and Hai1, which were mapped into the QTL intervals to confirm their introgressive origin. These findings will enhance our understanding of the transcriptional regulations of fiber development and promote the progress of precise gene-editing-assisted cotton breeding in the future.

## 2. Results

### 2.1. Phenotypic Evaluation and Statistical Analysis of Cotton Fiber Quality

**Figure 1:**
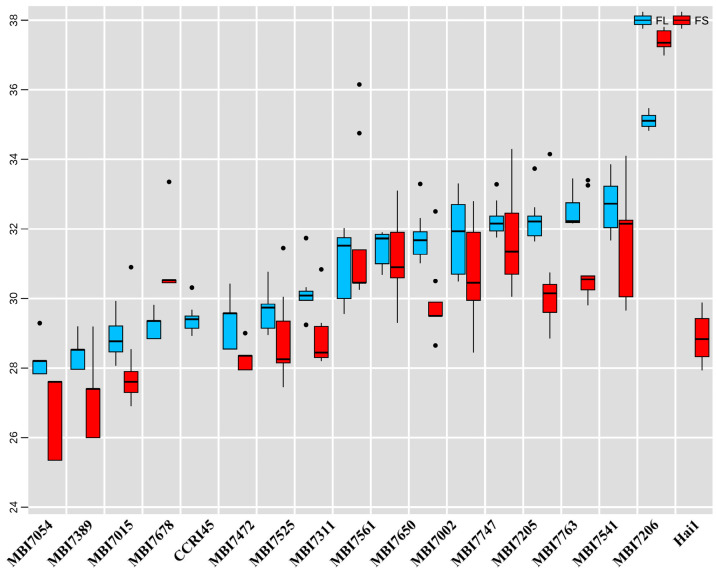
The performances of fiber length and strength tested in six environments within two years. The black point represented the outlier of phentypic data.

In order to obtain the comprehensive assessments on the fiber yield and quality traits, 15 CSSLs and their donor parent Hai1 and recurrent parent CCRI45 (also known as ZH45) were cultivated in different locations for two years, and a total of six environment data were collected, including Anyang, Henan Province (AY), Changde, Hunan Province (CD), Shangqiu, Henan Province (SQ), in 2012, and AY, CD, and Zhoukou, Henan Province (ZK) in 2013. The naturally opened mature balls sampled in the field were firstly subjected to indoor testing, and ≥15 g lint samples were sent to the Laboratory of Quality and Risk Assessment for Cotton Products (Institute of Cotton Research, Ministry of Agriculture) for fiber length (FL) and fiber strength (FS) evaluation. The statistical analysis is shown in Table S1, and the highest and lowest values of FL and FS traits were separately observed in Hai1 and MBI7054. The results showed that the FL and FS of MBI7054, MBI7389, MBI7015, MBI7678, MBI7472, MBI7525, and MBI7311 were lower than or close to those of CCRI45 and significantly lower than those of Hai1, while FL and FS of the other eight CSSLs were significantly higher than those of CCRI45 (Figure 1). Collectively, the 15 CSSLs exhibited relatively stable traits in fiber quality, with a certain degree of diverse variation compared with their parents, providing ideal materials for further research on transcriptome sequencing and introgressive events.

### 2.2. RNA-Seq, Enrichment Analyses, and WGCNA of FL Related DEGs

To systematically uncover key genes and pivotal signaling pathways involved in fiber elongation, developing fiber samples of 10 CSSLs that exhibited significant differences in FL performance, together with their parents Hai1 and CCRI45, were chosen to perform RNA-seq, each of which were sampled at 10 DPA with three biological repeats. Principal component analysis (PCA) of the RNA-seq data of these 36 fiber samples revealed that most of them could gather together, except MBI7002 and MBI206, which presented slightly scattered stats in the two dimensions (Figure S1). Pearson correlation coefficient (PCC) analysis of them indicated that over 93% and 87% of correlation coefficients were reached among the samples across three biological repeats (Figure S2). These results imply that the RNA-seq data were of high quality and eligible for further bioinformatic analyses.

**Figure 2:**
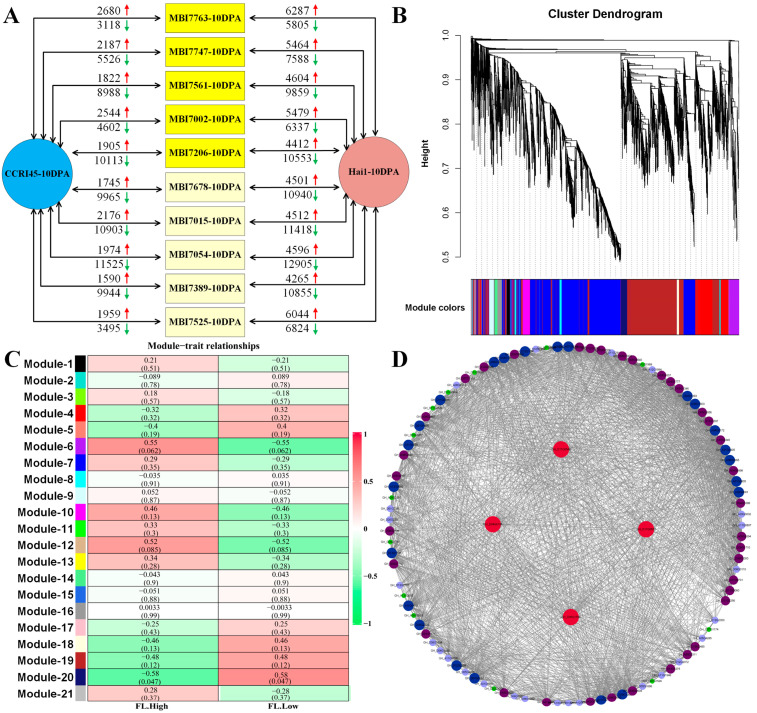
The DEG identification and co-expressed network analysis conducted on the FL-related RNA-seq. (**A**) The pairwise comparisons of FL-related samples for identifying DEGs. The red arrow represented the up-regulated DEG number, and the green arrow represented the down-regulated DEG number. (**B**) The cluster dendrogram of FL-related DEGs. (**C**) The heat map of the correlation between modules and traits. (**D**) The co-expression network analysis of FL-related DEGs.

Five FL-high CSSLs (namely MBI7763, MBI7747, MBI7561, MBI7002, and MBI7206) and five FL-low CSSLs (namely MBI7678, MBI7015, MBI7054, MBI7389, and MBI7525) were chosen to perform pairwise comparisons with their parents CCRI45 and Hai1, respectively (Figure 2A). The results indicated that the total number of observed DEGs was much higher than that between individual sample pairs, which was consistent with the facts that different CSSLs harbored different introgressed DNA fragments of *G. barbadense*, although these CSSLs were closer to the same genetic background after consecutive backcrossing with the recurrent parent CCRI45 for five or more generations. WGCNA of these DEGs by utilizing the dynamic pruning tree method with the combination of weight values and expressing similarities established a total of 21 modules (Figure 2B). The MEmidnightblue module was significantly correlated with FL performance (Figure 2C), which was composed of 112 DEGs. The biological process (BP) terms of the DEGs of the MEmidnightblue module included xyloglucan metabolic process (GO:0010411), hemicellulose metabolic process (GO:0010410), and cell wall polysaccharide metabolic process (GO:0010383); the molecular function (MF) terms included xyloglucan:xyloglucosyl transferase activity (GO:0016762), coenzyme binding (GO:0050662), and inositol-1,4-bisphosphate 1-phosphatase activity (GO:0004441); while the cellular component (CC) terms included apoplast (GO:0048046), extracellular region (GO:0005576), and cell wall (GO:0005618) (Figure S2A). A large number of FL-related DEGs were found to participate in the KEGG pathways of cutin, suberine and wax biosynthesis (ko00073), tryptophan metabolism (ko00380), pyruvate metabolism (ko00620), pentose phosphate pathway (ko00030), and fructose and mannose metabolism (ko00051, Figure S2B and Table S2). The co-expressed network of the 112 DEGs was visualized by utilizing a cytoscape in Figure 2D, and four hub DEGs were identified in the central nodes, namely *GH_D11G0546*, *GH_D04G0138*, *GH_D11G0652*, and *GH_D05G1798.*

**Figure 3:**
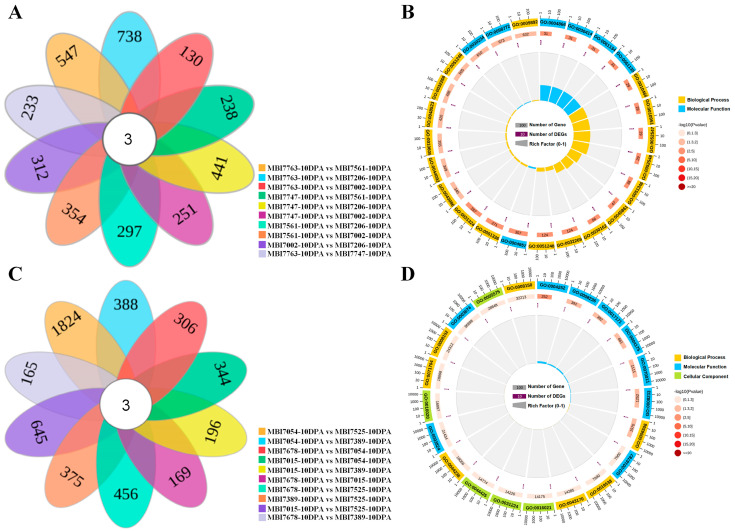
The common DEG identification and functional enrichment analysis of the FL-related DEGs. (**A**,**C**) The Venn diagrams of common DEG from the pairwise comparisons among the FL-high and FL-low CSSLs. (**B**,**D**) The GO enrichment analyses of common DEGs related with FL-high and FL-low traits.

In order to identify common DEGs among FL-high or FL-low CSSLs, pairwise comparisons were firstly conducted in each and every FL-high CSSL pair (Figure S3A). The results exhibited that the pairs of MBI7763-10DPA vs. MBI7206-10DPA/MBI7561-10DPA and MBI7747-10DPA vs. MBI7206-10DPA shared the top three largest number of down-regulated DEGs and the pairs of MBI7002-10DPA vs. MBI7747-10DPA/MBI7561-10DPA and MBI7206-10DPA vs. MBI7561-10DPA shared the top three largest number of up-regulated DEGs. The results of multiple comparisons of DEGs of FL-high CSSLs identified that three common ones, *GH_A01G2197*, *GH_D07G2083*, and *GH_scaffold3193_objG0001*, were shared by all these CSSLs (Figure 3A). GO enrichment analysis indicated the common DEGs were mainly related to negative regulation of peptidase activity (GO:0010466), negative regulation of endopeptidase activity (GO:0010951), and regulation of peptidase activity (GO:0052547) in BP terms; endopeptidase inhibitor activity (GO:0004866), peptidase inhibitor activity (GO:0030414), and peptidase regulator activity (GO:0061134) in MF terms; and integral component of membrane (GO:0016021), intrinsic component of membrane (GO:0031224), and membrane part (GO:0044425) in CC terms (Figure 3B). In pair-wise comparisons in FL-low CSSLs, however, the opposite phenomenon was observed, in that seven of the ten pairs shared more up-regulated DEGs than down-regulated DEGs (Figure S3B). Similarly, three common DEGs were shared across all five FL-low CSSLs, namely *GH_A02G0895*, *GH_A08G0463*, and *GH_D06G0919* (Figure 3C). The most significantly enriched GO terms of these were proteolysis (GO:0006508), protein metabolic process (GO:0019538), and macromolecule metabolic process (GO:0043170) in BP terms; serine-type endopeptidase activity (GO:0004252), serine-type peptidase activity (GO:0008236), and serine hydrolase activity (GO:0017171) in MF terms; and integral component of membrane (GO:0016021), intrinsic component of membrane (GO:0031224), and membrane part (GO:0044425) in CC terms (Figure 3D).

### 2.3. RNA-Seq, Enrichment Analyses, and WGCNA of FS Related DEGs

The top five FS-high CSSLs, including MBI7541, MBI7747, MBI7561, MBI7560, and MBI7205, and the bottom 5 FS-low CSSLs, including MBI7389, MBI7015, MBI7311, MBI7472, and MBI7054, together with their parents CCRI36 and Hai1, were chosen to perform FS-related transcriptome sequencing using developing fibers sampled at 20 DPA.. The PCA of these 36 RNA-seq data showed that they clustered into two dimensions (Figure S1C), and PCC analysis revealed no less than an 82% correlation coefficiency among all the samples (Figure S1D). These results implied the reliability of these RNA-seq data, which provided a solid foundation for further DEG identification, WGCNA, and functional enrichment analyses.

**Figure 4:**
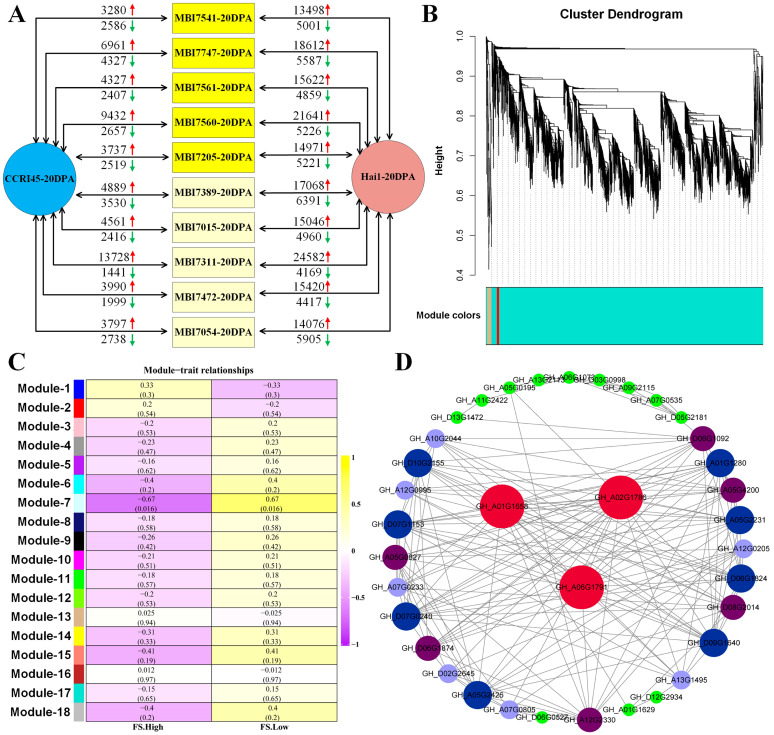
The DEG identification and co-expressed network analysis conducted on the FS-related RNA-seq. (**A**) The pairwise comparisons of FS-related samples for identifying DEGs. The red arrow represented the up-regulated DEG number, and the green arrow represented the down-regulated DEG number. (**B**) The cluster dendrogram of FS-related DEGs. (**C**) The heat map of the correlation between modules and traits. (**D**) The co-expression network analysis of FS-related DEGs.

The pairwise comparisons of each of the 10 CSSLs with CCRI45 and with Hai1, respectively, revealed more DEGs between each CSSL and Hai1 than between that CSSL and CCRI45 (Figure 4A). Furthermore, it also showed that there were more up-regulated DEGs than down-regulated ones in each and every pair between the CSSL and its parent, implying their potential significance for fiber development (Figure 4A). The WGCNA of these DEGs resulted in 18 expression modules by performing the dynamic pruning tree method (Figure 4B). The MElightcyan module was significantly correlated with the FS trait, which was composed of 43 annotated DEGs (Figure 4C). Subsequent functional enrichment analyses indicated that the top3 enriched GO terms (Figure S2C) were malate transmembrane transport (GO:0071423), negative regulation of ethylene-activated signaling pathway (GO:0010105), and negative regulation of phosphorelay signal transduction system (GO:0070298) in regard to BP terms; hydrolase activity, hydrolyzing O-glycosyl compounds (GO:0004553), hydrolase activity (GO:0016798), acting on glycosyl bonds, and malate transmembrane transporter activity (GO:0015140) in regard to MF terms; and membrane part (GO:0044425), protein phosphatase type 2A complex (GO:0000159), and protein serine/threonine phosphatase complex (GO:0008287) in regard to CC terms. The DEGs in the MElightcyan module were enriched the KEGG pathways of pyrimidine metabolism (ko00240), base excision repair (ko03410), MAPK signaling pathway–plant (ko04016), purine metabolism (ko00230), and the mRNA surveillance pathway (ko03015) (Figure S2D and Table S2). The co-expressed network analysis identified three hub DEGs, *GH_A01G1658*, *GH_A02G1786*, and *GH_A06G1791*, in the central nodes of the network, suggesting their potential contributions to fiber strength (Figure 4D).

**Figure 5:**
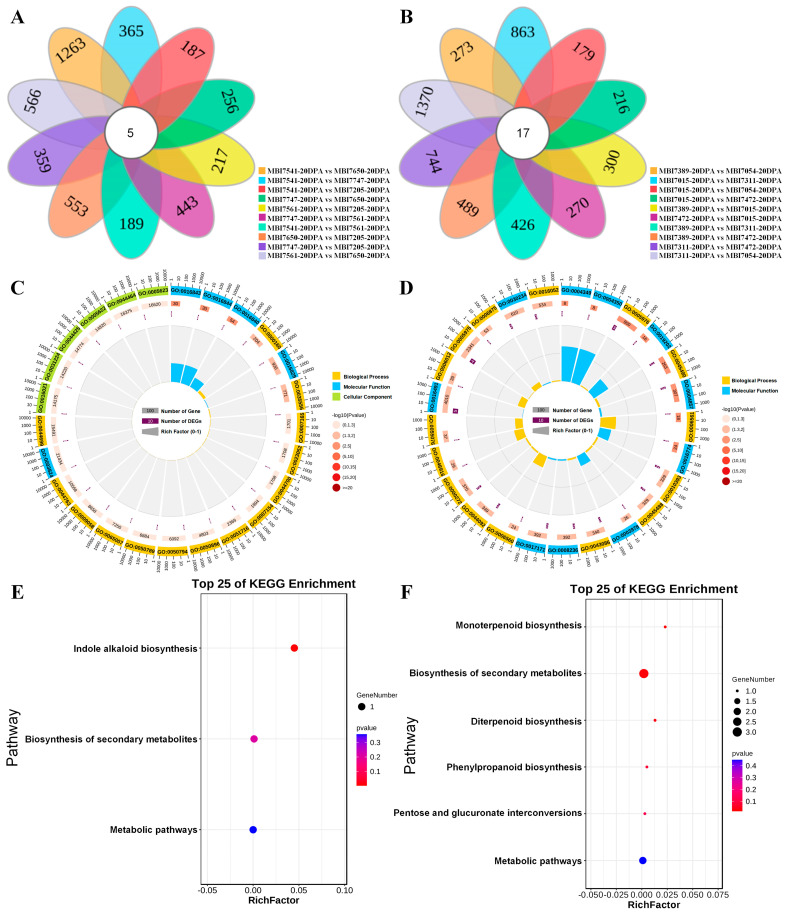
The common DEG identification and functional enrichment analysis of the FS-related DEGs. (**A**,**D**) The Venn diagrams of common DEGs from the pairwise comparisons among the FS-high and FS-low CSSLs. (**B**,**E**) The GO enrichment analyses of common DEGs related with FS-high and FS-low traits. (**C**,**F**) The KEGG enrichment analyses of common DEGs related with FS-high and FS-low traits.

In order to identify common DEGs among FS-high CSSLs, pairwise comparisons were conducted in each and every pair of the 5 FS-high CSSLs (Figure S3C). The results revealed that six of the ten pairs shared more up-regulated DEGs than down-regulated DEGs, except for the pairs of MBI7561-20DPA vs. MBI7747-20DPA, MBI7561-20DPA vs. MBI7205-20DPA, MBI7205-20DPA vs. MBI7747-20-DPA, and MBI7205-20DPA vs. MBI7650-20DPA. A total of five common DEGs were shared by all five FS-high CSSLs (Figure 5A), namely *GH_A07G2287*, *GH_A11G0783*, *GH_D02G2023*, *GH_D07G0784*, and *GH_D12G2043*. GO enrichment (Figure 5B) indicated that these common DEGs were significantly annotated in the phosphorelay signal transduction system (GO:0000160), intracellular signal transduction (GO:0035556), and signal transduction (GO:0007165) of the BP terms, which were found to be enriched in three KEGG pathways (Figure 5C), including indole alkaloid biosynthesis (ko00901), biosynthesis of secondary metabolites (ko01110), and metabolic pathways (ko01100). Similarly, pairwise comparisons of FS-low CSSLs revealed that the number of pairs, which shared more up-regulated DEGs, was the same as the number of pairs, which shared more down-regulated DEGs (Figure S3D). A total of 17 DEGs that were identified were across FS-low CSSLs (Figure 5D). These common DEGs were significantly enriched in the polysaccharide metabolic process (GO:0005976), pectin catabolic process (GO:0045490), and proline biosynthetic process (GO:0006561) of the BP terms (Figure 5E). These 17 common DEGs were annotated in six KEGG pathways (Figure 5F), including monoterpenoid biosynthesis (ko00902), biosynthesis of secondary metabolites (ko01110), diterpenoid biosynthesis (ko00904), phenylpropanoid biosynthesis (ko00940), pentose and glucuronate interconversions (ko00040), and metabolic pathways (ko01100). These results may help in dissecting the molecular mechanism of fiber strength formation.

### 2.4. RNA-Seq, Enrichment Analyses, and WGCNA of Fiber Quality-Related DEGs

A phenotypic evaluation of fiber quality exhibited that MBI7747 and MBI7561 simultaneously showed longer FLs and stronger FSs, while MBI7054, MBI7015, and MBI7389 simultaneously shorter FLs and weaker FSs. These five CSSLs, together with CCRI36 and Hai1, were selected to perform RNA-seq using developing fibers separately sampled at 10 and 20 DPA, resulting in a total of 42 fiber samples, including each with three biological repeats. PCA revealed that they tightly clustered in two dimensions (Figure S1E), and PCC analysis showed that their correlation coefficients reached from 80% to 93% (Figure S1F), suggesting the reliability of these RNA-seq data.

**Figure 6:**
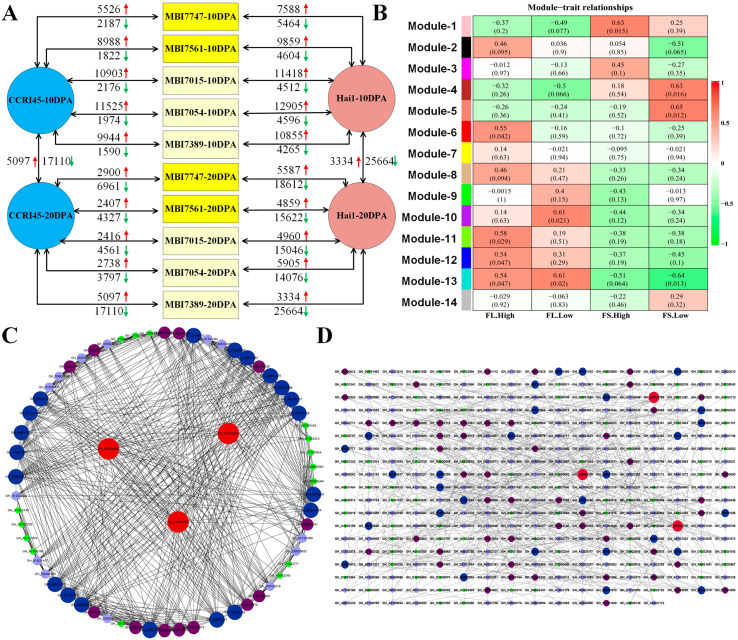
The DEG identification, functional enrichment, and co-expressed network analysis conducted on the FQ-related RNA-seq. (**A**) The pairwise comparisons of fiber development-related samples for identifying DEGs. The red arrow represented the up-regulated DEG number, and the green arrow represented the down-regulated DEG number. (**B**) The heat map of the correlation between modules and traits. (**C**) The co-expression network analysis of FL-high DEGs. (**D**) The co-expression network analysis of FS-high DEGs.

Subsequently, the DEGs between a CSSL and CCRI45/Hai1 at a fiber developmental stage were identified via pairwise comparisons between the two at that stage. Each of the five CSSLs was compared with both CCRI45 and Hai1, respectively, at both 10 DPA and 20 DPA separately (Figure 6A), and the fact that more DEGs were identified from the comparisons between CSSL and Hai1 was consistent with the results of FL-related and FS-related RNA-seq. A total of 17,619 DEGs were identified. It could be observed that more up-regulated DEGs than down-regulated ones were observed in all pairs at 10 DPA while opposite results were observed at 20 DPA. A GWCNA of these DEGs utilizing the dynamic pruning tree method with the combination of weight values and expressing similarities resulted in 14 network modules (Figure S4A). a Correlation coefficient between the module and the phenotypes revealed that the module MEgreenyellow was significantly correlated with FL-high, MEpink FS-high, MEpurple FL-low, and MEsalmom FS-low, respectively (Figure 6B). Enrichment analysis of the annotated DEGs of the modules MEgreenyellow and MEpink (totally 423 DEGs) revealed that the top3 GO terms (Figure S2E) were protein folding (GO:0006457), S-adenosylmethionine metabolic process (GO:0046500), and S-adenosylmethionine biosynthetic process (GO:0006556) in regard to BP terms; structural constituent of cytoskeleton (GO:0005200), methionine adenosyltransferase activity (GO:0004478), and unfolded protein binding (GO:0051082) in regard to MF terms; and microtubule (GO:0005874), supramolecular fiber (GO:0099512), and polymeric cytoskeletal fiber (GO:0099513) in regard to CC terms. The top five annotated KEGG pathways were protein processing in endoplasmic reticulum (ko04141), Phagosome (ko04145), Glycolysis/Gluconeogenesis (ko00010), pentose and glucuronate interconversions (ko00040), and steroid biosynthesis (ko00100) (Figure S2F and Table S2). The co-expressed network construction revealed that three DEGs, namely *GH_A03G0926*, *GH_A12G0021*, and *GH_A03G0302*, were located at the hub nodes in the FL-high DEG network (Figure 6C). Three DEGs, *GH_A01G0769*, *GH_A10G0309*, and *GH_D01G1706*, were located at the hub nodes in the FS-high DEG network (Figure 6D). The co-expressed networks of the FL-low and FS-low DEGs were also constructed, the former of which identified five hub DEGs (*GH_A06G1428*, *GH_D05G0801*, *GH_D02G2548*, *GH_A01G1599*, and *GH_D13G0252*) (Figure S4B) while the latter of which also identified five hub DEGs, including *GH_A06G1875*, *GH_D02G2249*, *GH_A03G1781*, *GH_D13G0454*, and *GH_A12G0262* (Figure S4C).

**Figure 7:**
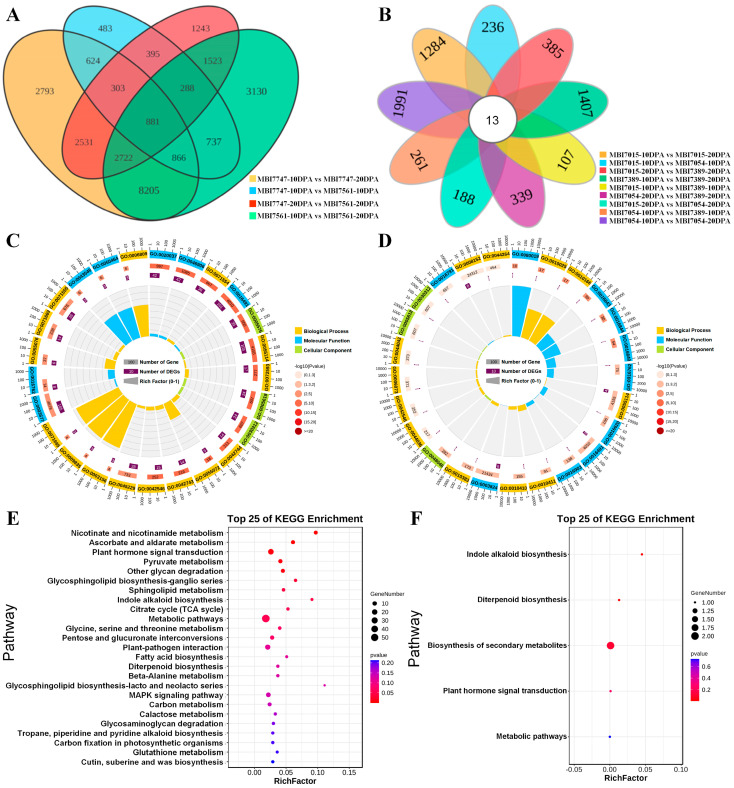
The common DEG identification and functional enrichment analysis of FQ-related DEGs. (**A**,**D**) The Venn diagrams of common DEG from the pairwise comparisons among the fiber quality-high and fiber quality-low CSSLs. (**B**,**E**) The GO enrichment analyses of common DEGs related with fiber quality-high and fiber quality-low traits. (**C,F**) The KEGG enrichment analyses of common DEGs related with fiber quality-high and fiber quality-low traits.

In addition, the pairwise comparisons between 10 DPA and 20 DPA were conducted on CSSLs of superior quality (Figure S3E). In total, 881 common DEGs were screened from the Venn diagram of the four groups (Figure 7A), whose GO enrichment terms (Figure 7B) included the BP terms of cell wall organization or biogenesis (GO:0071554), oxidation reduction process (GO:0055114), and reactive oxygen species metabolic process (GO:0072593); the MF terms of heme binding (GO:0020037), tetrapyrrole binding (GO:0046906), and oxidoreductase activity (GO:0016491); and the CC terms of extracellular region (GO:0005576), reactive oxygen species metabolic process (GO:0072593), and cell wall (GO:0005618). Those significantly enriched KEGG pathways (Figure 7C) were composed of nicotinate and nicotinamide metabolism (ko00760), ascorbate and aldarate metabolism (ko00053), plant hormone signal transduction (ko04075), pyruvate metabolism (ko00620), and other glycan degradation (ko00511). Likewise, the FL- and FS-low CSSLs were chosen to perform the 10 DPA and 20 DPA sample comparisons (Figure S3F), while only one comparison group showed the more up-regulated number, namely MBI7054-20DPA vs MBI7015-10DPA. All nine groups were utilized to create a Venn diagram to help in screening for potentially important DEGs (Figure 7D), and 13 common DEGs were identified for further functional enrichment analyses. Most of the common DEGs were found to significantly participate in the GO terms of BP, including wax biosynthetic process (GO:0010025), wax metabolic process (GO:0010025), and oxidation reduction process (GO:0055114) in regard to BP terms; fatty-acyl-CoA reductase (alcohol-forming) activity (GO:0080019), amine–lyase activity (GO:0016843), and strictosidine synthase activity (GO:0016844) in regard to MF terms; and apoplast (GO:0048046), cell wall (GO:0005618), and external encapsulating structure (GO:0030312) in regard to CC terms (Figure 7E). These DEGs were also enriched in the five KEGG pathways, including indole alkaloid biosynthesis (ko00901), diterpenoid biosynthesis (ko00904), biosynthesis of secondary metabolites (ko01110), plant hormone signal transduction (ko04075), and metabolic pathways (ko01100) (Figure 7F). All this valuable information lay a solid foundation for illustrating the molecular mechanism of fiber development.

### 2.5. Prediction and Enrichment Analysis of Introgressed DEGs in CSSLs

**Figure 8:**
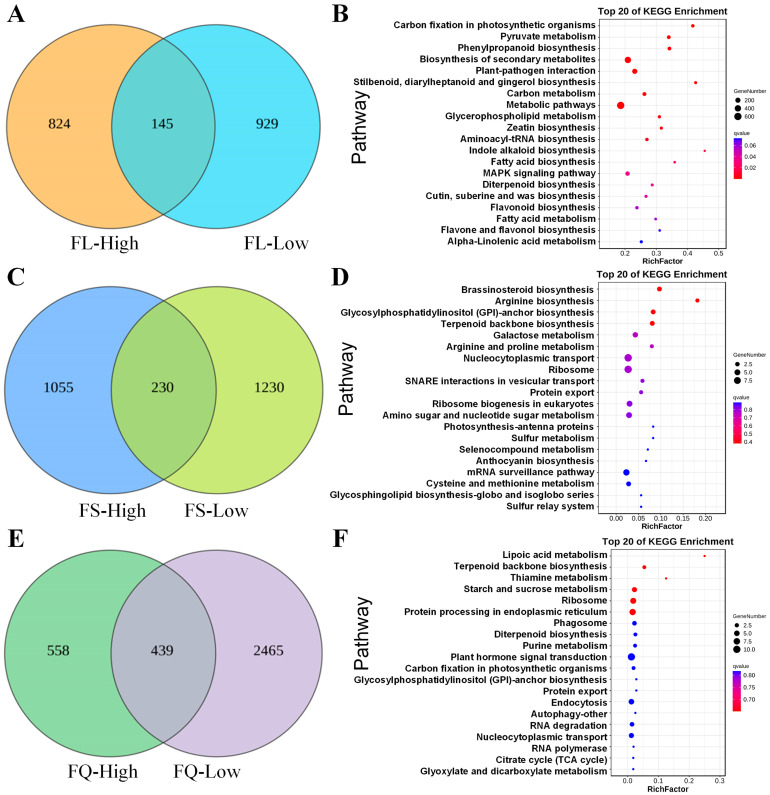
The introgressed prediction and functional enrichment analysis of DEGs relevant to FL, FS, and FQ traits in CSSLs. (**A**) The Venn diagram of introgressive DEGs in FL-related CSSLs. (**B**) The KEGG enrichment analysis of the introgressive DEGs in FL-high lines. (**C**) The Venn diagram of introgressive DEGs in FS-related CSSLs. (**D**) The KEGG enrichment analysis of the introgressive DEGs in FS-high lines. (**E**) The Venn diagram of introgressive DEGs in FQ-related CSSLs. (**F**) The KEGG enrichment analysis of the introgressive DEGs in FQ-high lines.

The introgressive gene from the donor parent might be the main reason for improving the traits of the receptor parent, which encouraged us to predict and identify the introgressive genes in CSSLs. Since the pairwise comparisons of the 15 CSSLs have been separately performed with CCRI45 and Hai1, all the DEGs between Hai1 and FL-high lines or FL-low lines were identified, which were regarded as the potential introgressive DEGs after removing the background DEGs between CCRI45 and the FL-high lines or FL-low lines. Therefore, a total of 969 and 1174 introgressive DEGs were predicted in FL-high and FL-low lines (Figure 8A), respectively. Of these, 145 DEGs were predicted to be common in both FL-high and FL-low lines. The significantly enriched GO terms of 824 DEGs that were specially expressed in FL-high linesincluded oxidation reduction process (GO:0055114), cell wall organization or biogenesis (GO:0071554), plant-type cell wall organization or biogenesis (GO:0071669) in regard to BP terms; heme binding (GO:0020037), tetrapyrrole binding (GO:0046906), and oxidoreductase activity (GO:0016491) in regard to MF terms; and intrinsic component of membrane (GO:0031224), integral component of membrane (GO:0016021), and extracellular region (GO:0005576) in regard to CC terms (Figure S4D). The significant enriched KEGG pathways of these DEGs were carbon fixation in photosynthetic organisms (ko00710), pyruvate metabolism (ko00620), phenylpropanoid biosynthesis (ko00940), biosynthesis of secondary metabolites (ko01110), and plant–pathogen interaction (ko04626) (Figure 8B).

Similarly, the FS-related introgressive DEGs were also predicted by pairwise comparing between Hai1 and FS-high or FL-low CSSLs and between CCRI45 and FS-high or FS-low CSSLs. A total of 1285 and 1460 putative introgressive DEGs were predicted to be relative to FS-high and FS-low CSSLs, respectively (Figure 8C). After removing 230 common DEGs shared by both FS-high and FS-low CSSLs, 1055 DEGs that were exclusively expressed in FS-high lines were subjected to functional enrichment analyses. The results indicated that the top3 enriched GO terms were response to carbohydrate (GO:0009743), response to glucose (GO:0009749), and cellular polysaccharide biosynthetic process (GO:0033692). The significantly enriched KEGG pathways of these DEGs were brassinosteroid biosynthesis (ko00905), arginine biosynthesis (ko00220), glycosylphosphatidylinositol (gpi)-anchor biosynthesis (ko00563), terpenoid backbone biosynthesis (ko00900), and galactose metabolism (ko00052) (Figure 8D).

The CSSLs with both FL-high and FS-high phenotypes (FQ-high) or with both FL-low and FS-low phenotypes (FQ-low) were also chosen to employ the prediction of introgressive DEGs (Figure 8E). A total of 997 and 1540 DEGs were identified in FQ-high and FQ-low CSSLs, respectively. The 588 DEGs that were exclusively expressed in FQ-high CSSLs were subjected to functional enrichment analyses in terms of the GO and KEGG pathways. The significantly annotated GO terms were protein localization (GO:0008104), purine ribonucleoside salvage (GO:0006166), and cellular protein localization (GO:0034613) in regard to BP terms; adenosine kinase activity (GO:0004001), Ran GTPase binding (GO:0008536), and Ras GTPase binding (GO:0017016) in regard to MF terms; and cell (GO:0005623), DNA-directed RNA polymerase IV complex (GO:0000418), and ribosome (GO:0005840) in regard to CC terms (Figure S4F). The significantly enriched KEGG pathways, in which these DEGs participated, were lipoic acid metabolism (ko00785), terpenoid backbone biosynthesis (ko00900), thiamine metabolism (ko00730), starch and sucrose metabolism (ko00500), and ribosome (ko03010) (Figure 8F).

### 2.6. Conjoint Analyses of FQ-Related QTL and Introgressive DEGs

Previous research on these CSSLs had reported FL- and FS-related QTL identification [25]. When the FL-related DEGs were aligned back to the *G*. *hirsutum* genome, 14 DEGs were successfully mapped into 11 of the 49 QTL intervals (Table 1). Sequence alignment analysis was conducted on these 14 DEGs with *G. hirsutum* and *G. barbadense* IDs and the results revealed that 12 of them showed a non-synonymous mutation in the protein sequences between their parental CCRI45 and Hai1, implying that these DEGs could be potential candidate genes responsible for FL development. Similarly, when the FS-related DEGs were aligned back to the *G*. *hirsutum* genome, 13 DEGs were successfully mapped into 8 of the 52 FS-related QTL intervals (Table 1). Sequence alignment analysis of these 13 DEGs with *G. hirsutum* and *G. barbadense* IDs indicated that 11 of them showed non-synonymous mutation in their protein sequences between their parental CCRI45 and Hai1, implying that these DEGs could be potential candidate genes responsible for FS development.

When the FQ-related DEGs were aligned back to the *G*. *hirsutum* genome, eleven DEGs were aligned into seven FL-related QTL intervals and 10 DEGs were related into 8 FS-related QTL intervals (Table 1). Sequence alignments analysis of the 21 DEGs with *G. hirsutum* and *G. barbadense* IDs indicated that eleven of them showed non-synonymous mutations in their proteins between their parental CCRI45 and Hai1.

## 3. Discussion

Cotton is an important economic crop that produces fiber for the textile industry and edible oil for the food industry [11]. Upland cotton (*Gossypium hirsutum*, *Gh*) features the advantages of wide adaption and high yield but also has ordinary fiber quality [25]. Varieties of sea island cotton (*G. barbadense*, *Gb*) are famous for their superior fiber quality [23]; therefore a potential solution for further improving upland cotton fiber quality is to introduce fine *Gb*-genes into the *Gh*-background [24]. The development of the CSSL population is usually derived from one cross between the donor parent and receptor parent with multi-generation backcrosses with the recurrent parent together with several generation selfing [27]. In consideration of harboring only a few introgressive segments or genes of the donor parent, the CSSLs are regarded as ideal materials for conducting QTL mapping on the agronomic traits in diverse crops [26,28,29,30]. In this study, CSSLs derived from CCRI45 and Hai1 in previous studies [25,31] were selected as materials, having been planted in the Yellow River and Yangtze River basins for two years. After performing the phenotypic analyses of cotton FL and FS (Figure 1 and Table S1), 10 FL-related CSSLs, including 5 FL-high and 5 FL-low ones, were selected for RNA-seq at 10 DPA. Similarly, 10 FS-related CSSLs, including 5 FS-high and 5 FS-low ones, were selected for RNA-seq at 20 DPA. Of these CSSLs, MBI7747 and MBI7561 showed both FL-high and FS-high phenotypes, while MBI7389, MBI7015, and MBI7311 showed both FL-low and FS-low phenotypes, collectively. In total, 15 CSSLs, together with their parents CCRI45 and Hai1, were selected to perform the current study.

More FL-related DEGs were observed between CSSLs and Hai1 than those between CSSLs and CCRI36, which were annotated in the biological processes of the xyloglucan metabolic process, hemicellulose metabolic process, and the cell wall polysaccharide metabolic process in the GO enrichment analysis, as well as in the KEGG pathways of cutin, suberine and wax biosynthesis, the pentose phosphate pathway, and fructose and mannose metabolism. Those results were consistent with the facts regarding fiber elongation formation involved in the interaction of celluloses, proteins, lipids, and sugar signaling molecules [14,32,33]. In addition, the hub DEGs screened via WGCNA, *GH_D11G0546* and *GH_D11G0652* separately encoded xyloglucan endotransglucosylase/hydrolase 7 (XTH7) and XTH5. Those XTH proteins were reported to participate in cell wall reconstruction [34,35]; their promoter regions contained plenty of growth and development-related cis elements. Another hub DEG, *GH_D04G0138*, encodes expansin A8 (EXPA8), which had been proven to contribute to cotton FL and FM [36]. The hub DEG, *GH_D05G1798*, belongs to the heavy metal transport/detoxification superfamily, which was reported to participate in the biological processes of fiber development and response to abiotic stress [37]. Meanwhile, common DEGs were separately screened via pairwise-comparison within the groups of FL-high and FL-low CSSLs (Figure 3). Interestingly, same number of common DEGs (three) were obtained in each of the groups. Only *GH_scaffold3193_objG0001* in the FL-high group was found to be involved in the regulation of peptidase or endopeptidase activity, while *GH_A02G0895* in the FL-low group was significantly enriched in the biological processes of proteolysis, protein metabolism, and macromolecule metabolism (Figure 3D). Additionally, there were no reports to illustrate the correlation between Kunitz family trypsin and protease inhibitor protein (*GH_scaffold3193_objG0001*) and fiber development. The FL-low *GH_A02G0895* belonged to the subtilase family, and the subtilases (SBTs) were found to participate in salt stress response by regulating hormone transport and cell wall repair [38].

Interestingly, more FS-related up-regulated DEGs were identified at 20 DPA than at 10 DPA, indicating that cotton plants might activate more positively responding genes to achieve the transition from fiber elongation to secondary wall thickening. The functional enrichment analysis of the 43 annotated DEGs in a WGCNA module that were significantly correlated to fiber strength indicated that they were enriched in the GO terms of malate transmembrane transport, negative regulation of ethylene-activated signaling pathway, and negative regulation of phosphorelay signal transduction system, as well as in the KEGG pathways of pyrimidine metabolism, MAPK signaling pathway, and purine metabolism. Previous studies have proven that the above biological processes could make contributions to fiber development [39,40,41,42]. Three hub DEGs screened via co-expressed network, *GH_A01G1658*, *GH_A02G1786*, and *GH_A06G1791*, encode WRKY DNA-binding protein 75, root hair specific 19, and tonoplast dicarboxylate transporter (TDT), respectively. The WRKY genes were reported not only to participate in adversity stress and disease resistance [43,44], but also to play crucial roles in fiber development [45]. The *TDT* genes can improve the malate content in tomatoes [46], and malate transmembrane transport is found to be involved in fiber development [39]. Moreover, among the five common DEGs screened by performing Venn analysis of all the FS-high CSSLs at 20 DPA, *GH_A07G2287* encoding response regulator 24 was found to participate in the GO terms of phosphorelay signal transduction system, intracellular signal transduction, and signal transduction, and *GH_D12G2043*, with the annotation of strictosidine synthase 2, was significantly enriched in the KEGG pathways of indole alkaloid biosynthesis, the biosynthesis of secondary metabolites, and the metabolic pathway (Figure 5). Despite the response, regulatory genes were reported to be involved in the regulation of abiotic stresses [47], which may make them potential candidate genes in regard to affecting fiber development. Similarly, most strictosidine synthase or strictosidine synthase-like (SS or SSL) genes were found to positively respond against abiotic and biotic stresses [48], while their relative indole alkaloid biosynthesis was significantly enriched in the fiber development under ABA treatment [49]. A total of 17 common DEGs were identified among all the FS-low CSSL samples, of which *GH_A03G0768* and *GH_A11G1185* were significantly enriched in the GO terms of polysaccharide metabolic and pectin catabolic processes and *GH_D07G1560* and *GH_D09G1479* mainly became involved in the KEGG pathways of monoterpenoid and diterpenoid biosynthesis. Either the polysaccharide metabolism or the terpenoid biosynthesis was proven to be closely correlated with secondary wall thickening or trichome development [50,51].

In the pair comparison among the CSSLs of both FL-high and FS-high phenotypes (MBI7561 and MBI7747) and the CSSLs of both FL-low and FS-low phenotypes (MBI7015, MBI7054, and MBI7389), there were more up-regulated DEGs than down-regulated DEGs at 10 DPA and more down-regulated DEGs than up-regulated ones at 20 DPA. This phenomenon might have something to do with the fiber developing stages. Functional enrichment analysis revealed that the KEGG pathways of glycolysis/gluconeogenesis, pentose and glucuronate interconversions, and steroid biosynthesis were proven to become involved in the fiber development [52,53,54]. Three hub DEGs relevant to FS-high phenotypes, *GH_A03G0926*, *GH_A12G0021*, and *GH_A03G0302* are annotated to the RING/U-box superfamily, RAC-like 2, and myosin-binding proteins, respectively, which have been reported to play important roles in fiber, root hair, and trichome development [55,56,57]. Most of the FQ-high DEGs were found to be significantly enriched in the biological processes of cell wall organization or biogenesis, ROS metabolism, phytohormone signal transduction, pyruvate metabolism, and glycan degradation, which have been proven to contribute to fiber development [1,17,58,59,60]. However, the common DEGs relevant to FQ-low phenotypes were also involved in the biological processes of wax biosynthesis or metabolism, oxidation reduction, IAA biosynthesis, and phytohormone signal transduction, all of which were found in previous studies to participate in fiber elongation and secondary wall thickness [11,61,62,63].

A total of 969 FL-high, 1285 FS-high, and 997 FQ-high DEGs were predicted to be introgressive, and we noticed that the most FL-high introgressive DEGs were involved in carbon fixation in photosynthetic organisms, pyruvate metabolism, and phenylpropanoid biosynthesis, while the biosynthetic pathways of brassinosteroid, arginine, and glycosylphosphatidylinositol (gpi)-anchor relevant to FS-high performance, as well as the metabolic pathways of lipolic acid, thiamine, and starch and sucrose related to FQ-high traits, were separately enriched. Collectively, the ROS metabolism, cell wall structure, carbohydrate metabolism, and phenylpropanoid metabolism were previously proven to make contributions to fiber elongation [52,64,65,66], and those metabolic processes that could affect secondary wall thickness were composed of glucose and polysaccharide biosynthesis and brassinosteroid and glycosylphosphatidylinositol metabolism [20,67,68,69]. Furthermore, the predicted introgressive DEGs were combined with the relative QTL in the CSSL populations constructed by CCRI45 and Hai1 [25], and we found that 12, 11, and 11 candidate genes showed a non-synonymous mutation between the *Gh* and *Gb* species in the 11 FL-related, eight FS-related, and 15 FQ-related QTL intervals (Figure 8). After intercomparions of all the candidate introgressive DEGs, only DEG was commonly expressed in the FL-related and FQ-related QTL intervals, namely *GH_D06G0090* encoding receptor-like protein kinase 1 (*RLK1*). Initially, leucine-rich repeat RLK proteins were found to participate in brassionsteroid (BR) signaling [70], and multiple recent studies indicated that BR signaling could make great contributions to fiber elongation [70,71,72,73,74,75,76]. The analyses of the relative gene family in *Gossypium* also indicated that the RLK and RLK1L proteins were not only involved in fiber development [77] but also play crucial roles in response to abiotic stresses [78]. Plenty of research has been conducted on the CSSL population by the methods of traditional genetic mapping and modern high-throughput multiomics, while only a few introgressive genes that were in control of fiber quality were fined mapped and verified [79,80], Therefore, those candidate introgressive DEGs in the QTL intervals undoubtedly lay the gene-pool foundations for further revealing the molecular mechanism of fiber development.

## 4. Materials and Methods

### 4.1. Cotton Materials and Phenotypic Statistics

The CSSL population was derived from a single hybridization between CCRI45 (as the recipient parent) and Hai1 (as the donor parent) in 2003 at the Institute of Cotton Research of the Chinese Academy of Agriculture Sciences (CRICAAS) (Anyang, China). The development procedure was detailed in a previous study [81]. The *G. hirsutum* variety CCRI45 was developed by CRICAAS, while the *G. barbadense* strain Hai1 was stored in our lab [82].

Having performed the phenotypic analyses of cotton FL and FS in 2012 in Anyang, Shangqiu (Henan Province), and Changde (Hunan Province); in 2013 in Anyang, Zhoukou (Henan Province), and Changde (Hunan Province) (Figure 1 and Table S1), 10 FL-related CSSLs, including 5 FL-high (MBI7763, MBI7002, MBI7561, MBI7206, and MBI7747) and 5 FL-low (MBI7525, MBI7054, MBI7015, MBI7389, and MBI7678) ones, were selected for RNA-seq at 10 DPA. Similarly, 10 FS-related CSSLs, including 5 FS-high (MBI7561, MBI7541, MBI7205, MBI7747, and MBI7650) and 5 FS-low (MBI7054, MBI7015, MBI7311, MBI7389, and MBI7472) CSSLs, were selected for RNA-seq at 20 DPA. Of these CSSLs, MBI7747 and MBI7561 showed both FL-high and FS-high phenotypes, while MBI7389, MBI7015, and MBI7311 showed both FL-low and FS-low phenotypes, collectively; 15 CSSLs, together with their parents CCRI45 and Hai1, were selected to perform the current study.

### 4.2. Plant Cultivation and Sample Collection

The selected 15 CSSLs and their parents (CCRI45 and Hai1) were planted in the experimental fields of Anyang city (Henan Province), each of which was designed in 6 line plots (line length of 8 m, line spacing of 0.8 m, and plant spacing of 0.25 m) in 2021. The anthesis day of flowers were tagged and denoted as 0 days post anthesis (DPA) [11]. The developing bolls were sampled at 10 DPA and 20 DPA; fiber samples were peeled off the ovules and stored in liquid nitrogen for quick-freezing, and then stored at −80 °C until the next step of the experiments [11].

### 4.3. RNA Extraction and Library Sequencing

Total RNAs were extracted using the RNA prep Pure Plant Kit (Tiangen, Beijing, China). The RNA quality was assessed through agarose gel electrophoresis to check the degradation or contamination, and the RNA integrity was evaluated using the Nano 6000 Assay Kit of the Bioanalyzer 2100 system (Agilent Technologies, Santa Clara, CA, USA). After quantification with the Qubit^®^ RNA Assay Kit in a Qubit^®^ 2.0 Fluorometer (Life Technologies, Carlsbad, CA, USA), approximately 3 μg of total RNA samples were used to prepare cDNA library with the NEB-Next^®^ Ultra™ RNA Library rep Kit for Illumina^®^ (NEB, San Diego, CA, USA) [81].

The 150–200 nt fragments were purified using the AMPure XP system (Beckman Coulter, Beverly, MA, USA) and then subjected to PCR amplification for enrichment and collection. Further purification of PCR products was performed with the AMPure XP system, and the library quality was assessed on the Agilent Bioanalyzer 2100 system. The TruSeq Cluster Kit v3-cBot-HS (Illumina) was used for clustering the index-coded samples with the cBot Cluster Generation System [81]. A total of 72 cDNA libraries were sequenced on a flow cell using the Illumina HiSeq™ 2500 sequencing platform.

### 4.4. Transcriptome Sequencing and Data Analysis

After processing with in-house Perl scripts, the raw data in FASTQ was converted into read sequences and the corresponding base qualities were recorded. Subsequently, low-quality reads (those with adapters, poly-N >10% or Q20 <20%) were filtered out to obtain clean data, from which the Q30 and GC content were calculated. Alignment was conducted using TopHat v2.0.12 software with clean reads aligned to the *G. hirsutum* TM-1 reference genome [83]. Genome data and gene model annotation files were obtained from the CottonGen database (http://www.cottongen.org). Expression levels of the genes were assessed by counting read numbers mapped to each gene using HTSeq v0.6.1, and the FPKM was utilized for quantifying gene expression levels based on gene length and mapped read counts. Differential gene expression analysis was performed using DESeq2 between two different samples (and by the edgeR package in R4.2.0 for comparisons between two samples), with the differentially expressed genes/transcripts set as the values of false discovery rate (FDR) being less than 0.05 and the absolute fold change being equal to or more than 2. The functional enrichment of the GO categories and KEGG pathways were analyzed using OmicShare (https://www.omicshare.com/tools/, accessed on 24 February 2023) [84].

### 4.5. Construction of Weight Gene Co-Expressed Network

The gene co-expression network was constructed using the WGCNA package in the R package, and the FPKM of genes was introduced into the co-expressed network analysis [50,85]. According to the dynamic changes in gene expression signal values, the co-expression relationship between genes was calculated, and the gene transcription regulation model was established to obtain the regulatory relationship and direction of inter-gene expression. In order to find all gene expression regulatory network models and key genes in one or more species at different developmental stages, the modules were obtained using automatic network building function blocks with default settings. The eigenvalue of each module was calculated and used to test the correlation with each material and trait. The gene co-expression network and identification gene module were constructed using the block wise Modules() function, and the parameter max Block Size was set to the default value of 5000 [86]. It was generally required that the number of samples is greater than 15, and the more samples, the better. In gene modules that are highly correlated with phenotypes and have significant gene significance (GS), genes with node degrees greater than 5 are regarded as hub genes, and the study of genes related to traits from hub genes can achieve twice the results with half the effort. The interaction network of hub genes within the gene module was constructed and visualized using Cytoscape software (https://github.com/cytoscape/cytoscape, accessed on 24 February 2023) [87]. In order to further analyze the function of the hub gene, GO and KEGG enrichment analysis was performed on genes in modules with closely related traits to further clarify the possible biological functions of the hub gene and preliminarily explore its potential mechanism of action.

### 4.6. Prediction of Introgressive DEGs in CSSLs

In order to predict the potentially introgressive DEGs in the selected CSSLs at transcriptome resolution, the screening procedure was designed as followed: At first, all the FL-related DEGs were separately collected via performing pairwise comparisons between Hai1 and each FL-high or FL-low CSSL (for example, Hai1-10DPA vs. MBI77470-10DPA, Hai1-10DPA vs. MBI7561-10DPA); meanwhile, the pairwise comparisons between CCRI45 and each FL-high or FL-low CSSLs were also compared to generate the background DEGs, such as CCRI45-10DPA vs. MBI7747010DPA, CCRI45-10DPA vs. MBI7561-10DPA. After removing the DEG duplication of the CCRI45 background, FS-high or FL-low DEGs were supposed to be only expressed in Hai1 and CSSLs, which were regarded as the potentially introgressive DEGs in CSSLs. The screening processes of FS-related and FQ-related intorgressive DEGs were the same as the above procedure in FL-related DEG screening. Subsequently, a Venn diagram was utilized to screen the DEGs that were specific to the superior trait, which were then subjected to functional enrichment analyses based on the GO and KEGG databases.

### 4.7. Combination of QTL Intervals and Introgressive DEGs in CSSLs

The QTL mapping was performed using the same CSSL population, from which the above 15 CSSLs of the current study were selected, and a total of 49 FL-related and 52 FS-related QTL were identified in a previous report [25]. The predicted introgressive DEGs were subsequently mapped to QTL intervals to identify the candidate genes of that QTL, which were then subjected to sequence difference alignment between the protein sequences of the upland and sea island cotton genomes; additionally, the existing non-synonymous mutation or InDel could provide the evidence of its introgressive origin.

## 5. Conclusions

Cotton is an important economic crop, and its fiber quality performance and value mainly depend on its FL and FS. In order to investigate the molecular mechanism of fiber elongation and secondary wall thickening, 24 fiber samples at 10 DPA and 20 DPA were separately collected from 15 CSSLs and their upland and sea island cotton parents, CCRI45 and Hai1, respectively, for transcriptome sequencing. Pairwise comparisons between each of the CSSLs and their parental lines were performed to identify their DEGs, and, eventually, FL-related, FS-related, and FQ-related DEGs, were identified, which were then subjected to WGCNA and functional enrichment analyses in regard to their GO terms and KEGG pathways. The hub DEGs were identified in the gene co-expressed networks in fiber elongation and secondary wall thickening, and most of them had been reported to play important roles in fiber development. The introgressive DEGs were also confirmed in the CSSLs in the combined analysis of previous QTL mapping results. These findings are valuable for advancing research on the formation of fiber quality, enhancing our understanding of the underlying developing mechanisms of cotton fiber.

## Figures and Tables

**Table 1:** The combination of QTL intervals and introgressive DEGs in CSSLs.

Trait	QTL	*G. hirsutum* ID	*G. barbadense* ID	Non-Synonymous/Synonymous Mutation or Same Sequences
Fiber Length	*qFL-C7-3*	*GH_A07G1073*	*GB_A07G1063*	Non-synonymous
		*GH_A07G1074*	*GB_A07G1064*	Non-synonymous
	*qFL-C12-5*	*GH_A12G1295*	*GB_A12G1365*	Non-synonymous
	*qFL-C13-1*	*GH_A13G0163*	*GB_A13G0168*	Same sequences
	*qFL-C13-2*	*GH_A13G0454*	*GB_A13G0460*	Non-synonymous
		*GH_A13G0460*	*GB_A13G0468*	Non-synonymous
	*qFL-C14-2*	*GH_D02G0665*	*GB_D02G0693*	Non-synonymous
	*qFL-C14-5*	*GH_D02G1983*	*GB_D02G2045*	Non-synonymous
	*qFL-C15-2*	*GH_D01G0849*	*GB_D01G0882*	Non-synonymous
	*qFL-C17-2*	*GH_D03G0331*	*GB_D03G0325*	Non-synonymous
	*qFL-C21-1*	*GH_D11G0612*	*GB_D11G0613*	Non-synonymous
	*qFL-C21-2*	*GH_D11G0693*	*GB_D11G0698*	Non-synonymous
		*GH_D11G0711*	*GB_D11G0717*	Synonymous
	*qFL-C21-4*	*GH_D11G2888*	*GB_D11G2913*	Non-synonymous
Fiber Strength	*qFS-C2-2*	*GH_A02G1378*	*GB_A02G1399*	Same sequences
	*qFS-C7-4*	*GH_A07G1076*	*GB_A07G1066*	Non-synonymous
		*GH_A07G1078*	*GB_A07G1068*	Non-synonymous
	*qFS-C15-1*	*GH_D01G0056*	*GB_D01G0054*	Non-synonymous
		*GH_D01G0067*	*GB_D01G0064*	Non-synonymous
		*GH_D01G0071*	*GB_D01G0068*	Non-synonymous
	*qFS-C15-4*	*GH_D01G0480*	*GB_D01G0498*	Non-synonymous
	*qFS-C19-1*	*GH_D05G0262*	*GB_D05G0265*	Non-synonymous
		*GH_D05G0264*	*GB_D05G0267*	Non-synonymous
	*qFS-C21-1*	*GH_D11G0616*	*GB_D11G0417*	Non-synonymous
	*qFS-C21-5*	*GH_D11G2944*	*GB_D11G2973*	Non-synonymous
	*qFS-C25-1*	*GH_D06G0090*	*GB_D06G0111*	Non-synonymous
		*GH_D06G0098*	*GB_D06G0118*	Synonymous
Fiber Quality	*qFL-C2-5*	*GH_A02G1372*	*GB_A02G1393*	Same sequences
	*qFS-C7-1*	*GH_A07G0378*	*GB_A07G0371*	Same sequences
		*GH_A07G0380*	*GB_A07G0373*	Same sequences
	*qFS-C7-2*	*GH_A07G0464*	*GB_A07G0458*	Same sequences
	*qFS-C9-1*	*GH_A09G1695*	*GB_A09G1819*	Non-synonymous
	*qFL-C12-5*	*GH_A12G1294*	*GB_A12G1364*	Same sequence
	*qFS-C13-1*	*GH_A13G0148*	*GB_D13G0149*	Non-synonymous
		*GH_A13G0165*	*GB_A13G0170*	Non-synonymous
	*qFL-C13-2*	*GH_A13G0456*	*GB_A13G0463*	Same sequences
	*qFL-C15-2*	*GH_D01G0853*	*GB_D01G0886*	Non-synonymous
	*qFS-C14-3*	*GH_D02G1927*	*GB_D02G1995*	Non-synonymous
	*qFL-C14-5*	*GH_D02G1984*	*GB_D02G2046*	Non-synonymous
	*qFS-C19-1*	*GH_D05G0268*	*GB_D05G0271*	Non-synonymous
	*qFL-C19-2*	*GH_D05G1491*	*GB_D05G1497*	Non-synonymous
		*GH_D05G1492*	*GB_D05G1498*	Synonymous
	*qFL-C21-2*	*GH_D11G0692*	*GB_D11G0697*	Non-synonymous
		*GH_D11G0696*	*GB_D11G0701*	Synonymous
		*GH_D11G0701*	*GB_D11G0706*	Synonymous
		*GH_D11G0711*	*GB_D11G0717*	Synonymous
	*qFS-C21-5*	*GH_D11G2942*	*GB_D11G2971*	Non-synonymous
	*qFS-C25-1*	*GH_D06G0090*	*GB_D06G0111*	Non-synonymous

## Data Availability

The original contributions presented in the study are included in the article, further inquiries can be directed to the corresponding author.

## Supplementary Materials

The following supporting information can be downloaded at: https://www.mdpi.com/article/10.3390/plants13162308/s1, Table S1: The phenotypic data of fiber length and strength of different cotton lines; Table S2: The phenotypic data of fiber length and strength of different cotton lines; Figure S1: The PCA and PCC results of fiber RNA-seq samles; Figure S2: The enrichment analyses of GO and KEGG pathway on the common DEGs; Figure S3: The pairwise comparison among the samples of FL-related, FS-related, and FQ-related lines; Figure S4: The WGCNA and enrichment analyses of FL-low and FS-low lines and DEGs.
