# Supplementary material for: Co-Expression Network Analysis and Introgressive Gene Identification for Fiber Length and Strength Reveal Transcriptional Differences in 15 Cotton Chromosome Substitution Segment Lines and Their Upland and Sea Island Parents
Source: Plants (Basel). 2024 Aug 19;13(16):2308. doi: 10.3390/plants13162308 (PMC11359254; doi:10.3390/plants13162308)
Supplement: Supplementary file 1 [file plants-13-02308-s001.zip › Table S1.pdf]

Table S1 The phenotypic data of fiber length and strength of different cotton lines

Lines	Years	Environments	Fiber Length	Fiber Strength
MBI7002	2012	Anyang	31.68	29.50
	2012	Changde	32.32	29.90
	2012	Shangqiu	31.27	28.65
	2013	Anyang	33.29	32.50
	2013	Changde	31.92	29.50
	2013	Zhoukou	31.01	30.50
MBI7015	2012	Anyang	28.77	27.90
	2012	Changde	29.64	26.90
	2012	Shangqiu	29.22	28.55
	2013	Anyang	29.94	30.90
	2013	Changde	28.47	27.30
	2013	Zhoukou	28.07	27.60
MBI7054	2012	Anyang	28.20	27.63
	2012	Changde	29.29	27.60
	2012	Shangqiu	27.84	25.35
MBI7025	2012	Anyang	32.16	31.35
	2012	Changde	31.75	30.70
	2012	Shangqiu	33.28	30.05
	2013	Anyang	32.37	34.30
	2013	Changde	32.82	34.20
	2013	Zhoukou	31.94	32.45
MBI7206	2012	Anyang	33.23	32.15
	2012	Changde	31.67	29.65
	2012	Shangqiu	33.86	30.05
	2013	Anyang	33.74	33.70
	2013	Changde	32.73	34.10
	2013	Zhoukou	32.04	32.25
MBI7311	2012	Anyang	30.09	28.45
	2012	Changde	30.21	28.20
	2012	Shangqiu	31.74	29.20
	2013	Anyang	30.32	30.84
	2013	Changde	29.95	28.30
	2013	Zhoukou	29.25	29.30
MBI7389	2012	Anyang	29.21	29.20
	2012	Changde	27.97	26.00
	2012	Shangqiu	28.53	27.40

Lines	Years	Environments	Fiber Length	Fiber Strength
MBI7472	2012	Anyang	28.55	28.35
	2012	Changde	29.58	29.00
	2012	Shangqiu	30.43	27.95
MBI7525	2012	Anyang	29.15	29.35
	2012	Changde	29.84	27.45
	2012	Shangqiu	30.77	28.15
	2013	Anyang	30.66	31.45
	2013	Changde	28.95	28.25
	2013	Zhoukou	29.74	30.05
MBI7541	2012	Anyang	32.16	30.65
	2012	Changde	32.19	29.80
	2012	Shangqiu	33.45	30.25
	2013	Anyang	32.88	33.40
	2013	Changde	32.23	33.25
	2013	Zhoukou	32.76	30.55
MBI7561	2012	Anyang	31.79	34.75
	2012	Changde	31.52	30.25
	2012	Shangqiu	31.75	30.45
	2013	Anyang	32.03	36.15
	2013	Changde	30.00	30.45
	2013	Zhoukou	29.56	31.40
MBI7650	2012	Anyang	31.00	30.90
	2012	Changde	30.68	29.30
	2012	Shangqiu	31.84	30.60
	2013	Anyang	31.86	33.10
	2013	Changde	31.73	32.35
	2013	Zhoukou	31.90	31.90
MBI7678	2013	Anyang	29.82	33.35
	2013	Changde	29.36	30.45
	2013	Zhoukou	28.85	30.53
MBI7747	2012	Anyang	30.49	31.90
	2012	Changde	31.93	28.45
	2012	Shangqiu	32.70	29.95
	2013	Anyang	33.31	32.80
	2013	Changde	30.70	30.45
	2013	Zhoukou	33.24	32.15

Lines	Years	Environments	Fiber Length	Fiber Strength
MBI7763	2012	Anyang	32.22	30.15
	2012	Changde	32.37	29.60
	2012	Shangqiu	32.62	28.85
	2013	Anyang	33.74	34.15
	2013	Changde	31.80	30.75
	2013	Zhoukou	31.64	30.40
CCRI45	2012	Anyang	28.92	28.83
	2012	Changde	29.50	27.93
	2012	Shangqiu	30.31	28.33
	2013	Anyang	29.68	29.89
	2013	Changde	29.15	29.64
	2013	Zhoukou	29.40	29.42
Hai1	2012	Anyang	34.82	37.72
	2012	Changde	35.11	36.98
	2012	Shangqiu	34.95	37.81
	2013	Anyang	35.33	37.35
	2013	Changde	35.47	37.24
	2013	Zhoukou	35.26	37.69
