# Supplementary material for: Co-Expression Network Analysis and Introgressive Gene Identification for Fiber Length and Strength Reveal Transcriptional Differences in 15 Cotton Chromosome Substitution Segment Lines and Their Upland and Sea Island Parents
Source: Plants (Basel). 2024 Aug 19;13(16):2308. doi: 10.3390/plants13162308 (PMC11359254; doi:10.3390/plants13162308)
Supplement: Supplementary file 1 [file plants-13-02308-s001.zip › Table S2.pdf]

Table S2 KEGG enrichment categories of DEGs selected by different strategies

Fiber traits	DEG numbers	Top5 KEGG pathways
Fiber length	112 DEGs	Ko00073<br>Cutin, suberine and wax biosynthesis<br>Ko00380<br>Tryptophan metabolism<br>Ko00620<br>Pyruvate metabolism<br>Ko00030<br>Pentose phosphate pathway<br>Ko00051<br>Fructose and mannose metabolism
Fiber strength	43 DEGs	Ko00240<br>Pyrimidine metabolism<br>Ko03410<br>Base excision repair<br>Ko04016<br>MAPK signaling pathway - plant<br>Ko0020<br>Purine metabolism<br>Ko03015<br>mRNA surveillance pathway
Fiber quality	423 DEGs	Ko04141<br>Protein processing in endoplasmic reticulum<br>Ko04145<br>Phagosome<br>Ko00010<br>Glycolysis / Gluconeogenesis<br>Ko00040<br>Pentose and glucuronate interconversions<br>Ko00100<br>Steroid biosynthesis
